# Label-Free Macroscopic Fluorescence Lifetime Imaging of Brain Tumors

**DOI:** 10.3389/fonc.2021.666059

**Published:** 2021-05-24

**Authors:** Maria Lukina, Konstantin Yashin, Elena E. Kiseleva, Anna Alekseeva, Varvara Dudenkova, Elena V. Zagaynova, Evgenia Bederina, Igor Medyanic, Wolfgang Becker, Deependra Mishra, Mikhail Berezin, Vladislav I. Shcheslavskiy, Marina Shirmanova

**Affiliations:** ^1^ Institute of Experimental Oncology and Biomedical Technologies, Privolzhsky Research Medical University, Nizhny Novgorod, Russia; ^2^ Department of Neuromorphology, Research Institute of Human Morphology, Moscow, Russia; ^3^ Lobachevsky State University of Nizhny Novgorod, Nizhny Novgorod, Russia; ^4^ Becker&Hickl GmbH, Berlin, Germany; ^5^ Department of Radiology, Washington University School of Medicine, St Louis, MO, United States

**Keywords:** fluorescence lifetime imaging, FLIM, rat glioma model, glioblastoma, autofluorescence, image processing

## Abstract

Advanced stage glioma is the most aggressive form of malignant brain tumors with a short survival time. Real-time pathology assisted, or image guided surgical procedures that eliminate tumors promise to improve the clinical outcome and prolong the lives of patients. Our work is focused on the development of a rapid and sensitive assay for intraoperative diagnostics of glioma and identification of optical markers essential for differentiation between tumors and healthy brain tissues. We utilized fluorescence lifetime imaging (FLIM) of endogenous fluorophores related to metabolism of the glioma from freshly excised brains tissues. Macroscopic time-resolved fluorescence images of three intracranial animal glioma models and surgical samples of patients’ glioblastoma together with the white matter have been collected. Several established and new algorithms were applied to identify the imaging markers of the tumors. We found that fluorescence lifetime parameters characteristic of the glioma provided background for differentiation between the tumors and intact brain tissues. All three rat tumor models demonstrated substantial differences between the malignant and normal tissue. Similarly, tumors from patients demonstrated statistically significant differences from the peritumoral white matter without infiltration. While the data and the analysis presented in this paper are preliminary and further investigation with a larger number of samples is required, the proposed approach based on the macroscopic FLIM has a high potential for diagnostics of glioma and evaluation of the surgical margins of gliomas.

## Introduction

Glioblastoma (Grade IV) is the most common and most aggressive form of malignant brain tumors with an overall survival of 14–15 months after complete surgical resection and adjuvant radiochemotherapy (1). Standard therapy for high-grade gliomas includes maximal safe surgical resection, followed by radiotherapy and chemotherapy. The largest possible surgical removal is recommended, while preserving neurological function. Since the extent of removal of pathological tissue defines, to a large degree, a prognosis of the disease, an accurate identification of the tumor margin during resection is crucially important (2).

During surgery, tumors are routinely identified based on the neurosurgeon’s experience, visual observation with white-light microscopy, and neuronavigation using intraoperative ultrasound-based data and/or preoperative MRI scan. Intraoperative histopathological analysis to estimate tumor infiltration is uncommon, in part, because it is laborious and time-consuming, and because it requires multiple samplings for conclusive diagnosis. However, the tumor margins cannot be properly defined with conventional imaging, especially with respect to the brain shift that inevitably occurs. Intraoperative fluorescence guidance with 5-aminolevulinic acid (5-ALA) that induces protoporphyrin IX fluorescence or exogenous contrast agents (e.g. sodium fluorescein or indocyanine green) has proven to be a useful technique to improve the resection, but the challenges associated with non-specific distribution of the fluorophore, timing and the subjective assessment of fluorescence still remain (3). In addition, the use of 5-ALA in most cases does not allow to detect low grade gliomas, although it seems to be possible based on the fluorescence lifetime information (4). To avoid non-specific distribution of the fluorophores, fluorescent reporters targeting epidermal growth factor (EGF) receptor or other tumor specific molecules have been reported (5, 6). However, the question about the apparent toxicity of exogenous contrast agents upon systemic administration was left open. Stain-free techniques that do not require sample staining is a preferable way for tumor identification.

In recent years, there is an increasing interest in using endogenous fluorescence for intraoperative assessment in glioma surgery (7). Several studies indicate that autofluorescence in the blue range, derived mainly from reduced nicotinamide adenine dinucleotide (phosphate) NAD(P)H (ex/em 330-380 nm/420-480 nm), has a great potential to differentiate between tumorous and normal brain tissues (8–12). NAD+/NADH is a principal redox couple in the reactions of cellular respiration, such as glycolysis, the tricarboxylic acid cycle (TCA) and oxidative phosphorylation. NADP+/NADPH participates in anabolic reactions such as biosynthesis of fatty acids and nucleotides and in detoxifying processes. Metabolic alterations that accompany tumor progression can result in the change of the parameters of NAD(P)H fluorescence, including emission intensity, spectrum profile and the lifetime, which has been widely documented for gliomas (13–15). Among the fluorescence-based techniques, fluorescence lifetime imaging (FLIM) has the advantage of being a largely independent from the fluorophore concentration in combination with high molecular specificity. In the case of NAD(P)H, FLIM is able to resolve these molecules in their free unbound (~0.3-0.5 ns) and enzyme-bound (~2-4.5 ns) states, associated primarily with cytosolic and mitochondrial processes, correspondingly. While a significant progress has been recently achieved in the use of one-photon FLIM instrument in neurosurgery (16) in general, the possibilities of FLIM of NAD(P)H in glioma diagnostics on the whole tumor scale have been poorly explored so far.

Recently, we developed a system for fluorescence lifetime imaging on a macro scale (macro-FLIM) with high sensitivity to a relatively weak tissue autofluorescence (17). The system is based on scanning of the beam over a large field of view (∼18 mm) and confocal detection of the fluorescence signal. Recording of the fluorescence lifetime is done by time-correlated single photon counting (TCSPC) technique (18). Combination of the confocal detection with the fluorescence lifetime imaging using TCSPC enables recording of relatively high (15 μm) spatial and temporal resolution image within a reasonably short recording time. A large field of view in the macro-FLIM opens the opportunity to visualize the distribution of endogenous fluorescence from the entire tumor in animal or from the patients’ tissues samples in a relatively short period of time of (ca. 2 min/scan). This high speed imaging makes the method attractive for intraoperative applications such as stain-free pathology or image guided margin detection.

The objective of the present work was to investigate whether glioma and healthy brain tissue from the animal models and humans present different signatures in terms of the autofluorescence lifetime in the spectral band of NAD(P)H. In this study, the FLIM images were recorded from freshly excised whole rat brains with different intracranial glioma models. In the patient populations, surgical samples were collected from glioblastoma and the white matter. Fluorescence data were analyzed by two approaches: 1) nonlinear curve-fitting of fluorescent decays and 2) unsupervised visualization techniques based on Principal Component Analysis (PCA) and Linear Unmixing (LU). All samples were subjected to histopathological evaluation by an unbiased expert to verify the results of the imaging.

## Materials and Methods

### Intracranial Rat Glioma Models

The study was performed on Wistar rat glioma models - glioma C6 (n=5), glioblastoma 101.8 (n=3) and anaplastic astrocytoma 10-17-2 (n=6) (all females, body weight 240 ± 15 g). Healthy rats without tumors (n=3, females) were served as control. C6 rat glioma cells were cultured in DMEM containing 10% FBS in CO_2_ incubator (5% CO_2_, 37°C, humidified atmosphere). Cells were trypsinized (0.25% trypsin) for 3 min, washed and re-suspended in DMEM to the concentration 5×10^8^ cells/mL. 1×10^6^ cells in 10 μL PBS were used for injection into the rat brain. Anaplastic astrocytoma 10-17-2 and glioblastoma 101.8 were obtained by inoculation of homogenized tumor tissue from donor rats (~10^6^ tumor cells in 10 μL PBS) into the brain (19). For cells implantation, animals were anesthetized with zoletil (12.5 mg/kg) and xylazine (1 mg/kg) and immobilized on a stereotaxic unit. The indicated amounts of cells were injected *via* the hole drilled in the scull 2 mm lateral and 2 mm posterior to the bregma into the right hemisphere of the brain at ~4 mm depth.

Animals were euthanized using an overdose of zoletil (Virbac SA, France) and rometar (Spofa, Czech Republic) on 15-20 day after tumor inoculation. The brains were gently removed and divided in two tissue blocks in sagittal plane using a scalpel. One of the blocks was analyzed by FLIM immediately and then fixed in 10% buffered formalin for histological examination.

All animal procedures were approved by the Ethical Committee of the Privolzhsky Research Medical University (PRMU).

### Patient’s Samples

Human brain specimens were obtained from the University Clinic at PRMU from 5 patients during the tumor resection. An informed written consent was obtained from all patients prior to the enrollment. The samples of the glioblastoma (WHO grade IV) (n=5) and the peritumoral white matter (n=6) were investigated with FLIM and standard histology. Three out of six samples of the white matter had a high degree of tumor cell infiltration and were categorized as “with infiltration.” Other samples with no or a few isolated discretely located tumor cells were categorized as “without infiltration.”

The tissue storage was performed according to a previously developed protocol to preserve the fluorescence lifetime of NAD(P)H (20). Immediately after the resection, a tissue sample of 0.6-0.8 mm^3^ was wrapped in gauze soaked in 10% solution of bovine serum albumin (BSA), placed in a sterile Petri dish and delivered to laboratory on ice. FLIM was performed within 1.5 hours after the resection. Clinicopathological characteristics of patients are given in **Table 1**.

**Table 1:** Clinicopathological characteristics of patients.

	Age	Sex	Grade	IDH-status	Localization		Samples
Patient 1	32	F	Grade IV	NOS	left temporal	recurrent	WM with infiltration, tumor, tumor
Patient 2	39	M	Grade IV	IDH-mutant	left parietal	recurrent	tumor, tumor, tumor
Patient 3	64	M	Grade IV	IDH-wildtype	left parietal	recurrent	WM with infiltration, WM with infiltration
Patient 4	60	M	Grade IV	NOS	left fronto-pariet-occipital	recurrent	WM without infiltration
Patient 5	49	M	Grade IV	IDH-wildtype	right fronto-temporal	newly diagnosed	WM without infiltration, WM without infiltration

### FLIM on the Macroscale

A confocal macro-FLIM system, described earlier in our work (17), was utilized to scan centimeter-sized objects of animal and human tissue. Fluorophores are excited by picosecond diode lasers that form an excitation spot in the image plane of about 15 μm. Freshly excised samples are scanned by placing them directly in the image plane of a confocal scan head. The image plane of the scan lens is brought in coincidence with the sample surface. As the galvo-mirrors change the beam angle the laser focus scans across the sample. The fluorescence signal produced by the sample is collimated by the scan lens, descanned by the galvo-mirrors, and separated from the excitation light by the dichroic beamsplitter. It is further separated into two spectral channels and focused into the pinholes. Light passing the pinholes is sent to HPM-100-40 hybrid detectors (Becker&Hickl GmbH, Germany). The photon pulses from the detectors are processed by two SPC-150 TCSPC FLIM modules (Becker & Hickl GmbH, Germany). The maximum diameter of the image area in the primary image plane of the scanner is about 18 mm.

The tissue autofluorescence was excited by a picosecond diode laser (BDL-375-SMN, Becker&Hickl) at the wavelength of 375 nm with the power incident on a sample of 18 μW. The wavelength of excitation was selected based on the absorption of NAD(P)H at this wavelength. The signal was registered in the spectral range determined by a bandpass filter 460/50 nm (Chroma, USA). Collection time was 120 s, which allowed to collect from 6300 to 9700 photons per decay curve.

### FLIM Data Processing

SPCImage software (Becker & Hickl GmbH, Germany) was used to process the FLIM data. A nonlinear least-square fit was used to derive the decay parameters from the decay data in the pixels. The fluorescence decay curves were fitted with a bi-exponential decay model providing a short and a long lifetime components (τ_1_ and τ_2_, respectively), and the relative amplitudes of the lifetime components (a1 and a2, where a_1_ + a_2_ = 100%). From these values the amplitude-weighted mean fluorescence lifetime (τ_m_ = a_1_ τ_1_ + a_2_ τ_2_) and the ratio of the amplitudes, a_1_/a_2_ were derived. The quality of the fit was evaluated by the χ_2_ value. For all data presented here χ_2_ was within the appropriate range of 0.8 – 1.2.

**Figure 1:**
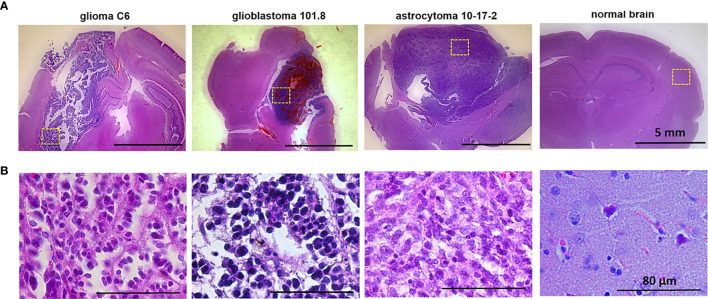
Histopathology of glioma models glioma C6, glioblastoma 101.8 and anaplastic astrocytoma 10-17-2 and normal rat brain. H&E-staining. **(A)** Initial magnification X7. **(B)** Initial magnification X40. Enlarged regions are indicated by the yellow squares on the lower-magnification panel. Bars are applicable to all images in the row.

**Figure 2:**
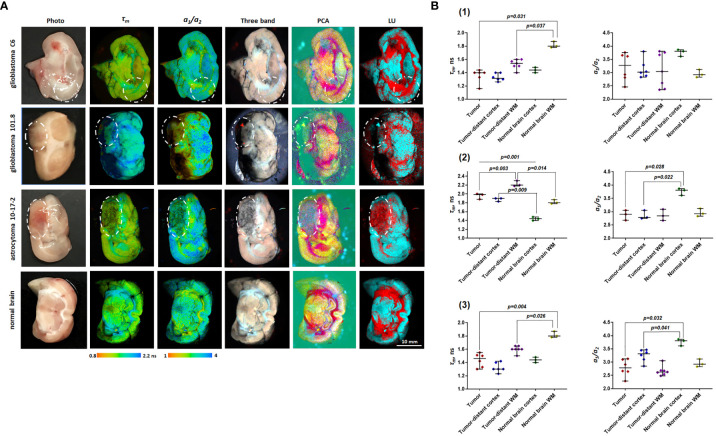
Macro-FLIM of glioma models and normal rat brain. **(A)** Representative autofluorescence time-resolved images of glioblastoma C6, glioblastoma 101.8, anaplastic astrocytoma 10-17-2 and rat brain without tumor. Pseudo RGB image is built from three time channels where the red color reflects fast emitting species, green medium, and blue long lived components; PCA images in the pseudo RGB images: red, green and blue colors correspond to a first, second, and third principal component; LU images reflect three selected classes: grey matter, white matter, and the background. The tumors are marked with a white line. Scale bar: 10 mm, applicable to all images. **(B)** Quantification of ***τ****_m_* and *a_1_/a_2_* ratio in (1) glioblastoma C6 (2), glioblastoma 101.8 (3), anaplastic astrocytoma 10-17-2 and normal brain. Scatter dot plot displays the measurements for individual animals (dots) and the median, minimum and maximum (horizontal lines). ***τ****_m_* is the mean fluorescence lifetime. *a_1_/a_2_* is the ratio of relative contributions of short and long components.

In each image of the rat brain, tumor areas and areas of cortex and white matter without tumor cells were selected as ROIs (**Figure S1**). Histograms of τ_m_ and, a_1_/a_2_ were calculated over the pixels of the ROIs. The maxima of the histograms were used to assess the tissue state in within the individual ROIs, see **Figures 1**, **2** and **Table 2**. Values derived from the maxima of the histograms have a much higher accuracy than data from single pixels. We were therefore ably to run the fits with all parameters, τ_1_, τ_2_, a_1_, a_2_, freely floating. This avoids biasing of the results by ignoring possible variations in the component lifetimes (21). For samples from human patients the same procedures were applied to the data from the entire sample.

**Table 2:** Autofluorescence lifetimes in patients’ glioblastoma and the peritumoral white matter.

Parameters			*τ_m_*, ns	*τ_1_*, ns	*τ_2_*, ns	*a*_*1*_, %	*a*_*2*_, %	*a*_*1*_*a*_*2*_
**Tumor**	Mean (SEM)		3.65 (0.17)	1.62* (0.05)	7.81* (0.27)	66.51* (1.46)	33.49* (1.46)	2.02* (0.15)
	Median		3.59	1.62	7.56	65.59	34.42	1.91
	Percentiles	25^th^	3.41	1.57	7.41	64.11	32.52	1.79
		75^th^	3.76	1.66	7.96	67.48	35.89	2.08
**Peritumoral WM with infiltration**	Mean (SEM)		4.06 (0.55)	1.71 (0.11)	8.35 (1.16)	63.31 (0.21)	36.69 (0.21)	1.73 (0.02)
	Median		4.35	1.71	9.08	63.25	36.75	1.72
	Percentiles	25^th^	3.67	1.62	7.58	63.12	36.53	1.71
		75^th^	4.59	1.79	9.49	63.47	36.89	1.74
**Peritumoral WM without infiltration**	Mean (SEM)		3.46 (0.35)	1.22 (0.05)	5.79 (0.51)	57.25 (0.89)	42.75 (0.89)	1.34 (0.05)
	Median		3.29	1.22	6.11	57.51	42.49	1.35
	Percentiles	25^th^	3.13	1.18	5.45	56.56	41.93	1.31
		75^th^	3.71	1.26	6.28	58.07	43.45	1.39

*p ≤ 0.05 from the peritumoral white matter without infiltration. Kruskal-Wallis test. n=3-5 samples per group. WT, white matter.

### Image Processing

FLIM collected datasets were analyzed using IDCube software (HSpeQ LLC, USA) that process 3D spectral data (22). FLIM raw data were first converted to the IDCube format and processed using a three- band method to generate a pseudo RGB image (23). The generated image is a composite of the three selected bands (time channels, i.e.) with the corresponding bandwidths, where the color intensity values are combined to form an RGB triplet. In this approach, we visualize the FLIM data with three colors where the Red, Green and Blue components were created by summing up the intensities values from three selected time ranges as shown in **Figure S2**, **Supplementary Information**. The data were then analyzed using Principal Component Analysis (PCA) that uses covariance matrices to compute association between data points. The most prominent association that accounts for most of the data variability is considered the first principal component, the second most variability is considered the second principal component, and so forth. The PCA was performed across all images and band selection was performed by selecting first two components that shows more than 80% of cumulative fraction of variance among 1024 components. In the PCA image three components were used assigned to Red, Green and Blue channels correspondingly. In that case, Cyan, Yellow and Magenta colors have been the result of mixing of Red, Green and Blue components.

Linear Unmixing (LU) was applied to classify the image and identify the regions of the tumors. This technique is used to determine the relative contribution from each fluorophore for every pixel of the image. The dataset was first processed by the N-FINDR method to estimate the 3 endmembers spectra, then least square method was used to generate the abundance maps based on the endmembers algorithm (24) to generate three endmembers. To estimate the optimal number of the endmembers, we calculated the residuals for the 2, 3 and 4 endmembers using nonnegative linear least-squares function available from MATLAB and implemented in IDCube. The residuals were calculated from the entire image and shown in **Figure S3**, **Supplementary Information**. As expected, we found the residual values were decreasing with the included number of endmembers, although the difference between the 3 and 4 endmembers seems to be marginal. We selected 3-endmember approach where the spectra resemble the decays representing the background, tumor and non-tumor tissues. The spectral characteristics of the endmembers are shown in **Figure S4**. The three endmembers resembled the long and short decays as well as the background. The two endmembers corresponding to the long and short decays were grouped together to form a pseudo RGB image. For the LU image we have used these two endmembers to visualize the image as pseudo RGB (Endmember #1 as Red, Endmember #2 as Blue, and as Green).

### Histopathology

Formalin-fixed brain samples were embedded in paraffin in accordance to a standard protocol and sectioned parallel to the optical plane. 7-μm thick paraffin sections were stained with hematoxylin and eosin and examined under microscopy with Axio Zoom.V16 (Zeiss, Germany) at x7 magnification and Leica DM2500 microscope (Leica, Japan) at x40 magnification.

### Statistics

The mean values, standard error of the mean (SEM), median, 25^th^ and 75^th^ percentiles were calculated for each quantitative parameter. To estimate the statistical significance of the differences between groups, the Kruskal-Wallis test was used. *P ≤* 0.05 indicated statistically significant difference.

## Results

### Histopathological Characterization of Rat Brain Tumors

Histopathological analysis of the rat brains *ex vivo* showed location of tumors inside the deep brain structures with the invasion into the white matter tracts and in the cortex (**Figure 1**).

At the cellular level, glioma C6 displayed typical characteristics of glioblastoma multiforme, such as high cellularity, regions of invasion into the brain parenchyma and the presence of necrotic areas. The tumors were composed of atypical cells with hyperchromic polymorphic nuclei. Numerous pathologic mitoses among proliferating cells were observed. Tumors were highly vascularized and presented a large number of newly formed microvessels. The clusters of proliferative cells were found preferentially along the vessels in the marginal zone of tumor.

Glioblastoma 101.8 at the advanced stage had a heterogeneous structure, large clusters of tumor cells alternated with necrotic areas and numerous small hemorrhages. Massive necrosis was observed in a central part of the tumor. Tumor margins were poorly defined, severe infiltration of the surrounding brain tissue with tumor cells was observed. The blood vessels were distributed heterogeneously with high variability of their diameters and irregular shapes. The tumor had high cellularity, high mitotic activity with a large number of pathological mitoses. Tumor cells had a high nuclear cytoplasmic ratio. The nuclei were pleomorphic, small or medium in size, with atypical arrangement of heterochromatin.

Anaplastic astrocytoma 10-17-2 composed of densely packed cells with hyperchromic polymorphic nuclei. A large amount of mitotic cells was detected, pathologic mitoses were rare. Necrosis was present in a moderate amount. The presence of peritumoral edema was revealed.

### Macro-FLIM of Rat Glioma Models

Time-resolved measurements of endogenous fluorescence from the tumorous and normal rat brain tissues showed that the fluorescence decays for all tissues were best fit to a double-exponential function with a short component τ_1_ ranged from 0.78 to 0.94 ns and a long component *τ_2_* ranged from 3.32 to 5.03 ns. One must mention that the measured fluorescence lifetimes were higher than those previously reported for NAD(P)H in cells and tissues: 0.3-0.5 ns for *τ_1_* (free NAD(P)H), and 2.0–3.2 ns for *τ_2_* (protein-bound NAD(P)H) (25–27), which indicates that any other fluorophores, likely, contribute to autofluorescence of the brain tissue.

In a search for differences between gliomas and normal brain tissue, we compared the parameters of fluorescence lifetime measurements (*τ_m_*, *τ_1_*, *τ_2_*, *a_1_*, *a_2_*, *a_1_/a_2_*) of the three rat glioma models with the cortex and the white matter, both tumor distant and intact control (**Table S1**).

Fluorescence lifetime values, such as *τ_m_*, in the C6 glioma region did not statistically differ from the tumor-distant cortex (1.46 ns vs 1.39 ns, *p*=1.0) and the tumor-distant white matter (1.46 ns vs 1.53 ns, *p*=0.176). However, these values were substantially lower than the corresponding values from the white matter of the healthy brain (*τ_m_* =1.46 ns vs 1.82 ns, *p* = 0.031; *τ_2_*= 3.39 ns vs 4.54 ns, *p* = 0.026). Lifetime parameters *τ_m_* and *τ_2_* in tumor-distant white matter were shorter compared to the white matter in healthy brain (*τ_m_* =1.53 ns vs 1.82 ns, *p*=0.037 and *τ_2_*= 3.55 vs 4.54 ns, *p* = 0.037, respectively) (**Figure 2 B1**).

For glioblastoma 101.8 we identified decreased *τ_m_* and *τ_2_* compared to tumor-distant white matter (*τ_m_* = 1.96 ns vs 2.23 ns, *p*=0.003; *τ_2 =_*4.61 ns vs 5.03 ns, *p*=0.042). In addition, all fluorescence lifetime parameters (*τ_m_, τ_1_, τ_2_, a_1_, a_2_, a_1_/a_2_*) in the tumor demonstrated differences from intact cortex (*p ≤* 0.043). Again, tumor-distant white matter and cortex differed from intact brain, showing longer lifetimes (*τ_m_, τ_1_, τ_2_*) (**Figure 2 B2**).

Anaplastic astrocytoma 10-17-2 displayed a reduced mean fluorescence lifetime in comparison with the intact white matter (*τ_m_* =1.51 ns vs 1.82 ns, *p*=0.004). Similar changes of tm were shown for the tumor-distant white matter compared with the intact control (1.61 ns vs 1.82 ns, *p*=0.014) (**Figure 2 B3**). Analysis of the amplitudes of the short (a1) and long (a2) lifetime components and their ratio (*a_1_/a_2_*) revealed an increase of a2 in the tumor and the tumor-distant cortex compared to the intact control cortex (~25 vs 20), resulting in a decreased *a_1_/a_2_*ratio (~3 vs 3.76).

Therefore, all three tumor models showed a difference between the tumor and the healthy brain tissue in, at least, one fluorescence lifetime parameter. The difference between the tumor and the tumor-distant brain tissues were detected only for glioblastoma 101.8. However, it was found that tumor-distant tissues also showed modified fluorescence lifetimes compared with healthy brain, indicating strong influence of the tumor on the surrounding area. These alterations in tumor-distant tissues could be associated with both diffuse infiltration of glioma cells in the brain and possible mechanical pressure of the tumor on the surrounding tissues, which subsequently results in the modification of cellular metabolism.

### Non-Fitting Approaches for FLIM Data From Rat Data

While fitting approach to the FLIM data results in a quantitative estimation of the decay parameters (fluorescence lifetimes and their relative amplitudes), it requires *a priori* knowledge about the number of fluorophores and an assumption that each fluorescent component presents a single decay. Although these methods are related to the actual physical characteristics of the fluorophores and considered to be gold standard, they require sufficient photon budget to accurately resolve the number and distribution of decay species from the data. This can be achieved with long acquisition times, which is not always appropriate for *in vivo* imaging and for effective translation of FLIM technique for clinical use. Non-fitting approaches offer another way to analyze FLIM data. These approaches rely on the datacube processing methods commonly used in hyperspectral imaging. FLIM generates a three dimensional dataset where in the addition to the two spatial coordinates, each pixel present the decay information forming a 3^rd^ dimension. It is similar to hyperspectral imaging where in addition to the spatial coordinate, each pixel present a spectrum. Thus, the methods developed for hyperspectral imaging can be applied to FLIM data. Specifically, we applied commonly used principal component analysis (PCA) and Linear Unmixing techniques for differentiation of the tumor from the tumor-distant white matter and cortex, and from the healthy brain without tumor. While both methods provide a good contrast to visualize astrocytoma in rats compare to the nearby tissues (**Figure 2**), in the Linear Unmixing the tumor appears in the same group as the corpus callosum. However, the methods were not sufficient to identify glioblastoma in rats.

### Macro-FLIM of Patient’s Glioblastoma

**Figure 3:**
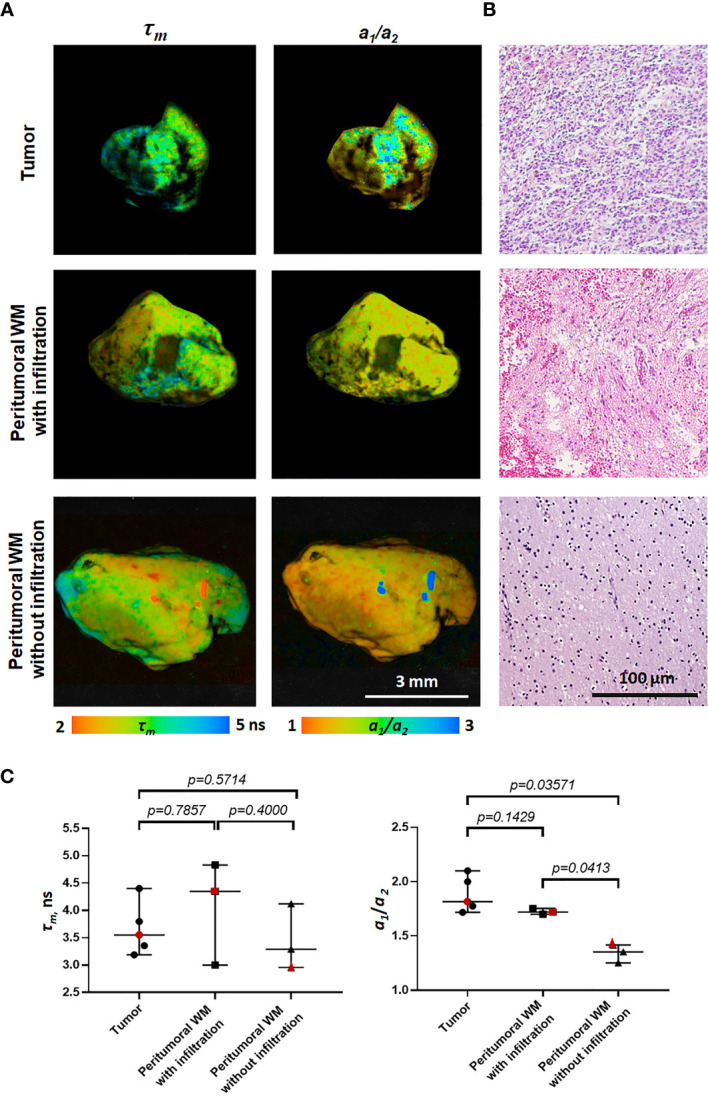
Macro-FLIM of patients’ glioblastoma and the peritumoral white matter with and without infiltration by tumor cells. **(A)** Representative autofluorescence time-resolved images. Scale bar: 3 mm. **(B)** Histopathology of samples shown in **(A)**. H&E-staining. Scale bar: 100 µm. **(C)** Quantification of *τ_m_* and *a_1_/a_2_* ratio in tumors and the peritumoral white matter. Scatter dot plot displays the measurements for individual samples (dots) and the median, minimum and maximum (horizontal lines). The values for the images in **(A)** are marked in red. *τ_m_* is the mean fluorescence lifetime. *a_1_/a_2_* is the ratio of relative contributions of short and long components.

FLIM analysis of fresh samples of glioblastoma (WHO grade IV) and the peritumoral white matter taken from patients after surgical resection showed fluorescence lifetimes of *τ_1_* ~1.6 ns and *τ_2_* ~7.8 ns (**Table 2**), which were even longer than the values in the rat brain. The fluorescence lifetimes of the short (*τ_1_*) and long (*τ_2_*) components were similar in the tumors and the white matter infiltrated by tumor cells, while in the noninfiltrated white matter both *τ_1_* and *τ_2_* were shorter (*τ_1_*~1.2 ns and *τ_2_*~5.7 ns). A comparison of the relative contributions *a_1_* and *a_1_* between tumors and normal tissue showed a statistically significant higher *a_1_* value (65.59% vs. 57.25%, *p* = 0.0413) and *a_1_/a_2_* ratio in the tumors in comparison with the white matter without infiltration (2.02 vs 1.34, *p* = 0.0413) with a high heterogeneity between glioblastoma samples (**Figure 3**). We should note that the infiltrated white matter did not differ in *a_1_/a_2_* ratio from the tumor samples.

**Figure 4:**
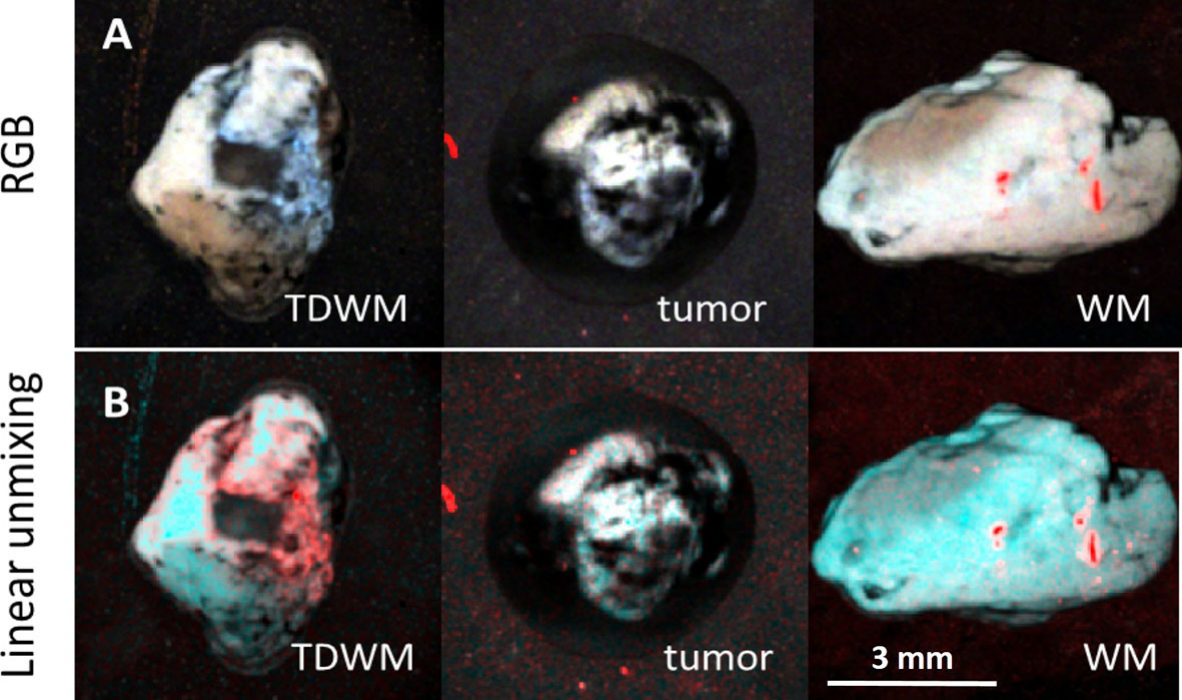
Image processing of 3D datasets from FLIM imaging of a peritumoral white matter with infiltration (TDWM), tumor and a white matter without tumor infiltration. **(A)** Three band pseudo RGB of the excised samples. **(B)** The image was processed Linear Unmixing with no supervision. Linear Unmixing of the datasets demonstrate a significant visual difference between the infiltrated white mater and the white matter. Scale bar: 3 mm.

The difference between the tumor and the peritumoral white matter can be also illustrated by Linear Unmixing of the samples (**Figure 4**). For that, corresponding FLIM datasets were open in the same window and three ROIs were selected as classes. One class corresponded to the tumor, another to the white matter, and third to the background. The endmembers spectra were automatically estimated using the 3-endmember approach over the entire image. The estimated spectra were used to classify the image and identify the tissue and the background. In the Linear Unmixing results, each class represented a specific color (red, green and blue). Strong visual difference between the tumor and the white matter indicates a significant distinction between two tissues, supporting the conclusions drawn from the fluorescence decay analysis.

## Discussion

There are several endogenous fluorophores that are involved in metabolic activity of tissues. The most important in the brain are NAD(P)H and flavins. Other fluorophores like tryptophan, tyrosine, various porphyrins, collagen and lipopigments are also present in brain tissue contributing to the autofluorescence (28, 29).

The attempts to evaluate whether the fluorescence lifetime of endogenous fluorophores, can be used to distinguish between healthy brain and brain tumors have been made in several previous studies (10, 28–32). A few studies demonstrate elongation of the NAD(P)H mean lifetime in brain tumor compared with the healthy tissue, which has been explained by the larger contribution of the bound form of the coenzyme to the fluorescence. For example, this was shown by Leppert et al. on G-112 human glioblastoma xenografts in mice, and by Kantelhardt et al. on U87 human glioma xenografts and human brain tumors using two-photon FLIM microscopy (12, 31). Interestingly, in the latter study the mean lifetimes decreased in the order glioblastoma> anaplastic glioma> low-grade astrocytoma>tumor-adjacent brain. Sun et al. and Marcu et al. detected higher values of the mean fluorescence lifetime in human glioblastoma multiforme *ex vivo* samples and intraoperationally in patients (33, 34). This is consistent with our observations of a longer mean lifetime in glioblastoma 101.8 compared with unaffected cortex. At the same time, several studies showed no statistically significant differences in the NAD(P)H fluorescence lifetime between glioma and the healthy brain tissue. These results were reported by Zanello et al. for freshly extracted human sample of gliomas using multimodal microscopic set-up (35) and by Haidar et al. on rats glioma RG2 *ex vivo* using a fiber-optical fluorescence probe (36). In our study, patients’ glioma samples did not differ from the peritumoral white matter in terms of the mean lifetime, while the *a_1_/a_2_* differed between the tumor and non-infiltrated white matter.

In the above mentioned and our studies, the mean fluorescence lifetime detected in NAD(P)H spectral range was significantly longer in brain tissues, including tumors, compared to cells and tissues of other localizations (20, 25, 35, 37). While for the latter ones, the mean fluorescence lifetimes were around 0.8-1.0 ns with the short and long lifetime components of around 0.3-0.5 ns, and 2.0-3.0 ns, respectively, for the former ones we measured the mean fluorescence lifetimes around 1.4-1.6 ns (animals, **Table S1**) and 3.4-4.5 ns (patient samples, **Table 2**).

The origin of the long fluorescence lifetimes of the signal where typically NAD(P)H autofluorescence is detected, is not clear so far. The phosphorylated form of NADH that has a rather long fluorescence lifetime (~4.4 ns) could contribute to the longer fluorescence lifetimes, since autofluorescence from NADH and NADPH are spectrally almost identical. However, such long lifetimes as 5.7-9.0 ns cannot be explained only with the contribution of NADPH. In addition, in brain tissue NADPH is widely believed to contribute minimally to the fluorescence signal, (38–40).

Collagens have a long fluorescence lifetime of around 5 ns and can in principle contribute to the autofluorescence signal in the range from 435 nm to 485 nm (41). However, their content in the brain is low (42).

The long fluorescence lifetimes observed in the NAD(P)H spectral window might stem from the fact that NAD(P)H itself, when it is bound to certain proteins may have the fluorescence lifetimes around 6-7 ns (43, 44).

Another factor that can lead to the anomalously long fluorescence lifetime in the brain is the presence of myelin. Myelin consists of 30% proteins and 70% lipids (45). Tyrosine and tryptophan are major aminoacids that constitute myelin sheath but their excitation bands are around 280 nm, which is far away from the excitation wavelength for NAD(P)H. Therefore, only lipids with the excitation wavelengths around 340-400 nm and detection in the spectral range of 400-500 nm may be responsible for the long fluorescence lifetime. Indeed, cholesterol, one of the major components of lipids, is known to have long fluorescence lifetimes on the order of 9 ns (46, 47). Moreover, time-resolved measurements of fluorescence from phospholipids, another major component of lipids, showed fluorescence lifetimes in the range from 7.0 to 12.0 ns, depending on the microenvironment (48). Regarding the possible contribution of myelin to the autofluorescence signal in the NAD(P)H spectral window elongation of the fluorescence lifetime for the patients’ samples compared with rats’ samples maybe explained by the higher content of myelin in the human brain. Indeed, while myelin in rats has a thickness of around 100-200 nm, human myelin typically reach 300 - 400 nm thickness (49). With the overall same total length of the myelin fibers in the rat and human brain, the volume of myelin in the human brain is almost twice higher than in the rat. More studies are needed to understand the nature of the long fluorescence lifetime in the brain, and this task can be better addressed with a standard FLIM microscope.

When comparing the fluorescence lifetime parameters of the tumor-distant brain in tumor-bearing animals with intact (healthy) brain tissues, we noticed that the former resemble more gliomas than the intact control. These data indicate that the tumor affects the biochemical state of surrounding tissue. For the rat models, it is no surprise that healthy brain tissues in the tumor-bearing animals are affected because the size of the tumor is large, so both the infiltration of the tumor cells in the brain and the mechanical pressure of the tumor on the surrounding tissues are inevitable (4, 50, 51).

The FLIM data obtained from the patients’ excised tissue (**Table 2**) showed similarity of the fluorescence decay parameters between glioblastoma and the white matter infiltrated by tumor cells. It is important that the tumor differed from the non-infiltrated white matter, which testifies to a potential of the method to differentiate between the tumor and the normal tissue in clinical settings. More insights into the influence of glioblastoma on peritumoral areas and correlation of fluorescence lifetimes with the degree of infiltration of adjacent tissue by tumor cells could be obtained with the use of samples extracted at the different distances from the tumor and by comparison with the brain samples from the non-tumorous patients.

Such controversial data across species point to the complexity of the malignant brain tumor behavior and/or lack of the reliable computational techniques to process FLIM data that restrict the use of FLIM technology in clinical oncology. Moreover, while the autofluorescence signals investigated in the previous works mentioned above (13, 30, 31, 33–35) were attributed only to NAD(P)H, there were no clear evidence that other fluorescent endogenous molecules did not make a contribution to the registered signal. In addition, direct comparison of the data obtained by different authors is challenging because of different experimental settings. Some groups consider control healthy tissue from the same brain but located outside of the tumor, while others consider controls as a tissue from a different brain that did not contain tumor. Standardization of the FLIM methods is required to have this tool accepted for clinical applications. The differences in the fluorescence decay parameters between the tumor and normal tissues require deep investigations with more samples and the use of molecular and biochemical techniques. 

To conclude, the obtained results of this pilot study with the use of macro-FLIM and endogenous fluorescence in the blue spectral range demonstrate that high-grade brain tumors have fluorescence lifetime signatures different from normal brain tissue and these differences can be visualized at the macroscale using 375 nm laser excitation. Taking into account a large number of fluorophores present in the brain tissue, the interrogation of the differences between tumor and normal tissue can be performed using alternative excitation and detection spectral ranges. This will be a future direction of our work. Both fitting and non-fitting methods of data analysis lead to the same conclusions about the status of the brain tissue. Non-fitting minimally supervised methods may be more relevant for clinical applications due the significant increase in the computational speed and thus possibility to use them for real time processing of the data. Ideally, the described non-fitting minimally supervised methods and other computational approaches should be used in clinical setting by the surgery or pathology teams without any prior knowledge of the tissue. However, the proposed computational methods need to be further validated with a larger number of tissues to identify a set of reliable features (markers) that reflect complex patterns of the tumors. One of the limitations of our study is that the ROIs were defined based on the gross observation prior the analysis. Future work will be focused on the tumor delineation using machine learning algorithms where the predictor model is based on the fluorescence lifetime imaging data combined with histological analysis. Overall, our preliminary results indicate that the new approach could find use in the clinic as a sensitive and accurate method for identifying the edges of tumors during surgery.

## Data Availability Statement

The original contributions presented in the study are included in the article/**Supplementary Material**. Further inquiries can be directed to the corresponding authors.

## Ethics Statement

The studies involving human participants were reviewed and approved by Ethics Committee of the Privolzhsky Research Medical University (Nizhny Novgorod, Russia). Written informed consent for participation was not required for this study in accordance with the national legislation and the institutional requirements. The animal study was reviewed and approved by Ethics Committee of the Privolzhsky Research Medical University (Nizhny Novgorod, Russia). Protocol #6 from April 17, 2019.

## Author Contributions

ML, EK, AA, and VD collected the data. ML, VS, MD, MB, and WB analyzed and interpreted FLIM data. EB, KY, and IM examined all pathology. MS and EZ coordinated and supervised the experiments. ML, MB, VS, and MS wrote the manuscript. All authors contributed to the article and approved the submitted version.

## Funding

The study has been supported by the Russian Foundation for Basic Research (project #18-29-01022). MB thanks NSF Awards PFI-TT 1827656 and NIH Award R01 CA208623.

## Conflict of Interest

MB is the founder of HSpeQ LLC, a hyperspectral imaging company that develops IDCube software. WB and VS was employed by the company Becker&Hickl GmbH.

The remaining authors declare that the research was conducted in the absence of any commercial or financial relationships that could be construed as a potential conflict of interest.
