# Supplementary material for: Label-Free Macroscopic Fluorescence Lifetime Imaging of Brain Tumors
Source: Front Oncol. 2021 May 24;11:666059. doi: 10.3389/fonc.2021.666059 (PMC8181388; doi:10.3389/fonc.2021.666059)
Supplement: Supplementary file 1 [file DataSheet_1.docx]

SUPPLEMENATRY INFORMATION

Label-free macroscopic fluorescence lifetime imaging of brain tumors

**Maria Lukina^1^, Konstantin Yashin^1^, Elena Kiseleva^1^, Anna Alekseeva^2^, Varvara Dudenkova^1^, Elena Zagaynova^3,1^, Evgenia Bederina^1^, Igor Medyanic^1^, Wolfgang Becker^4^, Deependra Mishra^5^, Mikhail Y. Berezin^5^, Vladislav Shcheslavskiy^4,1*^, Marina Shirmanova^1*^**

^1^Institute of Experimental Oncology and Biomedical Technologies, Privolzhsky Research Medical University, Nizhny Novgorod, Russia

^2^ Research Institute of Human Morphology, Moscow, Russia

^3^ Lobachevsky State University of Nizhny Novgorod, Nizhny Novgorod, Russia

^4^ Becker&Hickl GmbH, Berlin, Germany

^5^ Department of Radiology, Washington University School of Medicine, MO, USA


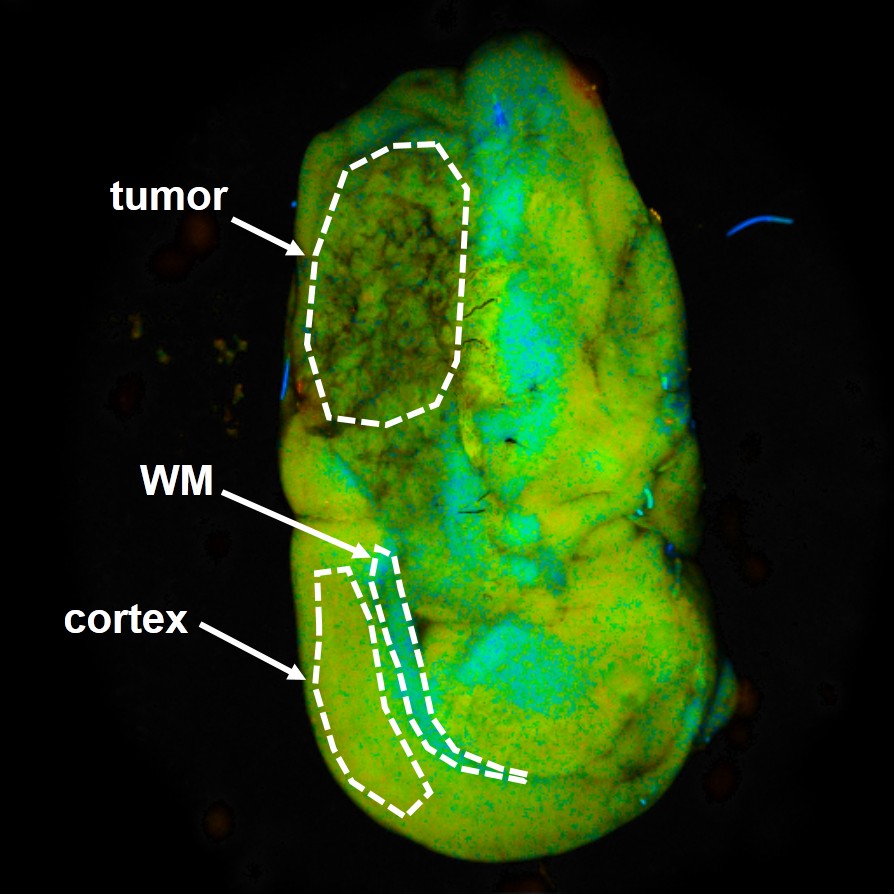


**Figure S1.** Selection of ROIs on the fluorescence lifetime image of the rat brain with tumor.


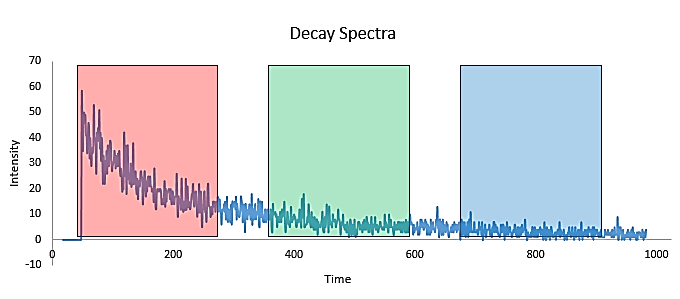


**Figure S2.** The principle of the pseudo RGB image obtained from fluorescence lifetime data


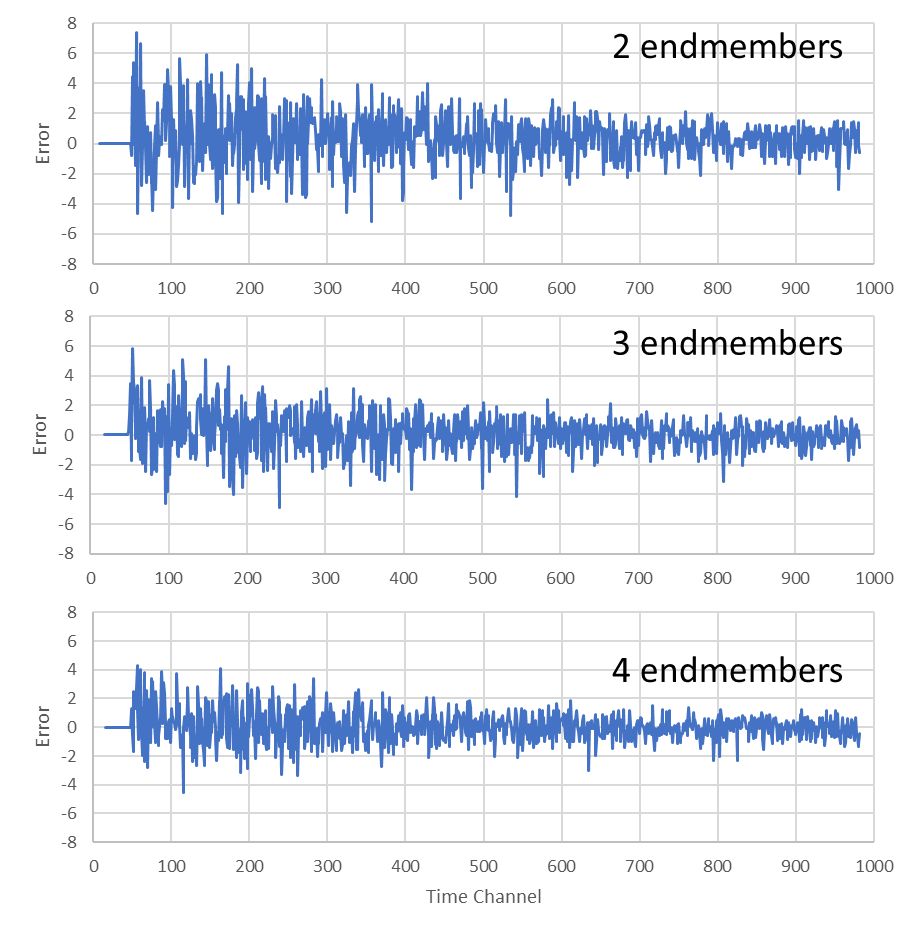


**Figure S3.** Calculated residuals for different number of endmembers.

| 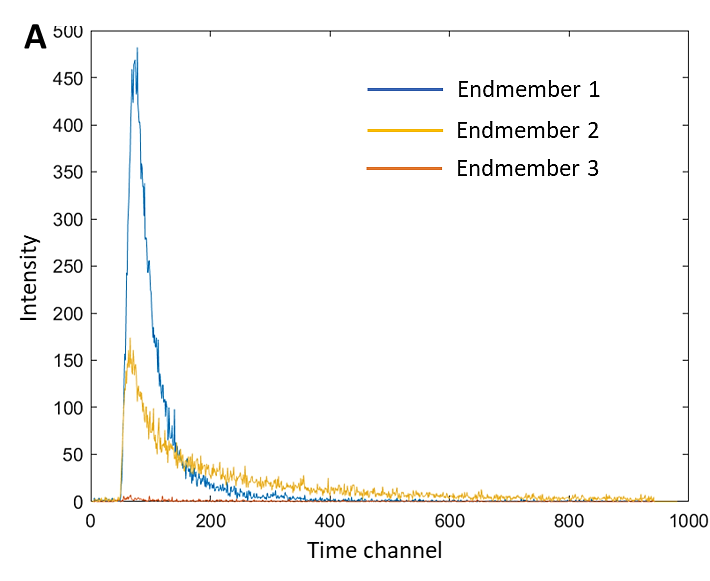 | 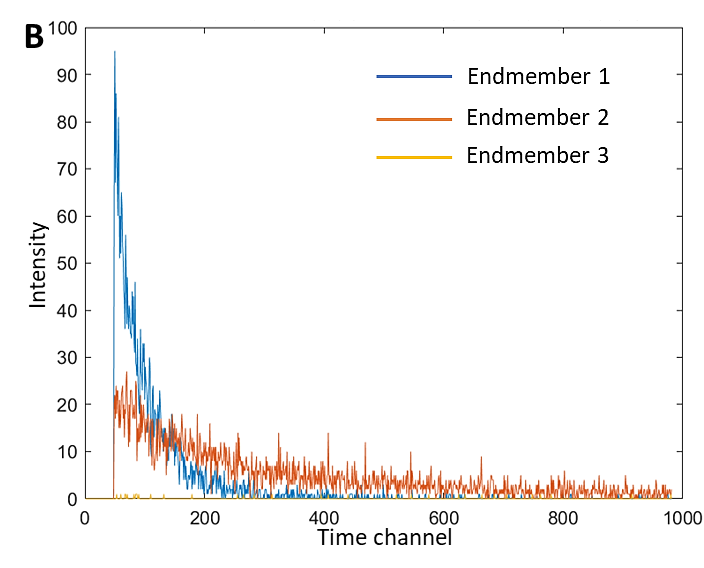 |
| --- | --- |
| 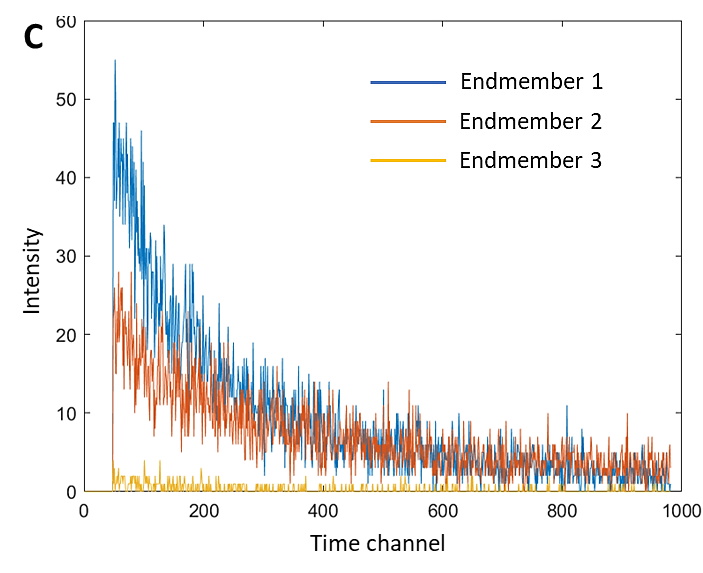  **Figure S4.** Endmember spectra calculated using N-FINDR method implemented in IDCube. (**A**): Tumor; (**B**): White matter with tumor infiltration; (**C**): White matter without tumor infiltration | |

**Table S1.** Autofluorescence lifetimes in rat gliomas and normal brain tissue.

|  | | | | ***τ_m_*, ns** | ***τ_1_*, ns** | ***τ_2_*, ns** | ***a_1_*, %** | ***a_2_*, %** | ***a_1_/a_2_*** |
| --- | --- | --- | --- | --- | --- | --- | --- | --- | --- |
| **glioblastoma C6** | Tumor | Mean (SEM) | | **1.46** (0.12) *** | 0.88 (0.09) | **3.39** (0.31) *** | 76.42 (1.32) | 23.58 (1.32) | 3.31 (0.23) |
|  |  | Median | | 1.4 | 0.78 | 3.2 | 78.34 | 21.66 | 3.62 |
|  |  | Percentiles | 25^th^ | 1.35 | 0.76 | 3.02 | 74.18 | 21.24 | 2.87 |
|  |  |  | 75^th^ | 1.4 | 0.82 | 3.52 | 78.76 | 25.83 | 3.71 |
|  | Cortex | Mean (SEM) | | 1.39 (0.07) | 0.78 (0.08) | 3.41 (0.19) | 76.21 (0.89) | 23.79 (0.89) | 3.24 (0.17) |
|  |  | Median | | 1.32 | 0.69 | 3.2 | 75.25 | 24.75 | 3.04 |
|  |  | Percentiles | 25^th^ | 1.3 | 0.68 | 3.03 | 74.63 | 22.08 | 2.94 |
|  |  |  | 75^th^ | 1.38 | 0.75 | 3.49 | 77.93 | 25.37 | 3.54 |
|  | WM | Mean (SEM) | | **1.53** (0.03) *** | 0.84 (0.05) | 3.55 (0.14) | 75.01 (1.65) | 24.99 (1.65) | 3.09 (0.27) |
|  |  | Median | | 1.54 | 0.84 | 3.44 | 75.29 | 24.72 | 3.05 |
|  |  | Percentiles | 25^th^ | 1.5 | 0.75 | 3.3 | 71.38 | 21.2 | 2.49 |
|  |  |  | 75^th^ | 1.59 | 0.94 | 3.86 | 78.8 | 28.62 | 3.72 |
| **glioblastoma 101.8** | Tumor | Mean (SEM) | | **1.96** (0.04)  *, ** | **1.02** (0.04)  ** | **4.61** (0.06)  *, ** | **74.16** (0.74)  ** | **25.84** (0.74)  ** | **2.88** (0.11)  ** |
|  |  | Median | | 1.98 | 1 | 4.63 | 74.37 | 25.63 | 2.9 |
|  |  | Percentiles | 25^th^ | 1.93 | 0.99 | 4.56 | 73.58 | 25.16 | 2.79 |
|  |  |  | 75^th^ | 1.99 | 1.03 | 4.66 | 74.84 | 26.42 | 2.98 |
|  | Cortex | Mean (SEM) | | **1.88** (0.02)  ** | **1.01** (0.05)  ** | 4.43 (0.09) | **74.06** (0.63)  ** | **25.94** (0.63)  ** | **2.86** (0.09)  ** |
|  |  | Median | | 1.9 | 1 | 4.45 | 73.56 | 26.44 | 2.78 |
|  |  | Percentiles | 25^th^ | 1.87 | 0.97 | 4.35 | 73.44 | 25.56 | 2.76 |
|  |  |  | 75^th^ | 1.9 | 1.05 | 4.52 | 74.44 | 26.57 | 2.92 |
|  | WM | Mean (SEM) | | **2.23** (0.04) *** | **1.19** (0.04) *** | 5.03 (0.12) | 74.08 (0.82) | 25.92 (0.82) | 2.87 (0.12) |
|  |  | Median | | 2.2 | 1.2 | 5.1 | 73.97 | 26.03 | 2.84 |
|  |  | Percentiles | 25^th^ | 2.19 | 1.15 | 4.95 | 73.35 | 25.24 | 2.75 |
|  |  |  | 75^th^ | 2.25 | 1.21 | 5.15 | 74.76 | 26.65 | 2.97 |
| **anaplastic astrocytoma 10-17-2** | Tumor | Mean (SEM) | | **1.51** (0.04) *** | 0.87 (0.04) | 3.56 (0.22) | **74.49** (0.86)  ** | **25.51** (0.86)  ** | **2.97** (0.12)  ** |
|  |  | Median | | 1.46 | 0.85 | 3.47 | 73.57 | 26.43 | 2.79 |
|  |  | Percentiles | 25^th^ | 1.35 | 0.76 | 3.12 | 72.62 | 24.88 | 2.65 |
|  |  |  | 75^th^ | 1.51 | 0.9 | 3.59 | 75.12 | 27.39 | 3.06 |
|  | Cortex | Mean (SEM) | | 1.36 (0.07) | 0.79 (0.03) | 3.32 (0.05) | 75.53 (0.52) | 24.47 (0.52) | **3.12** (0.09)  ** |
|  |  | Median | | 1.3 | 0.77 | 3.15 | 76.82 | 23.18 | 3.31 |
|  |  | Percentiles | 25^th^ | 1.3 | 0.72 | 3.1 | 75.86 | 22.59 | 3.14 |
|  |  |  | 75^th^ | 1.38 | 0.79 | 3.33 | 77.41 | 24.14 | 3.43 |
|  | WM | Mean (SEM) | | **1.61** (0.02) *** | 0.88 (0.02) | 3.55 (0.04) | 72.69 (0.53) | 27.31 (0.54) | 2.67 (0.08) |
|  |  | Median | | 1.6 | 0.89 | 3.53 | 72.44 | 27.56 | 2.63 |
|  |  | Percentiles | 25^th^ | 1.6 | 0.88 | 3.5 | 72.05 | 27.16 | 2.58 |
|  |  |  | 75^th^ | 1.63 | 0.89 | 3.62 | 72.84 | 27.96 | 2.68 |
| **normal brain** | Cortex | Mean (SEM) | | 1.44 (0.02) | 0.83 (0.02) | 3.91 (0.04) | 78.99 (0.34) | 21.01 (0.34) | 3.76 (0.08) |
|  |  | Median | | 1.44 | 0.82 | 3.89 | 79.2 | 20.8 | 3.81 |
|  |  | Percentiles | 25^th^ | 1.42 | 0.8 | 3.87 | 78.76 | 20.68 | 3.71 |
|  |  |  | 75^th^ | 1.46 | 0.84 | 3.95 | 79.33 | 21.24 | 3.84 |
|  | WM | Mean (SEM) | | 1.82 (0.03) | 0.94 (0.02) | 4.54 (0.07) | 74.7 (0.53) | 25.29 (0.53) | 2.96 (0.08) |
|  |  | Median | | 1.8 | 0.95 | 4.5 | 74.51 | 25.49 | 2.92 |
|  |  | Percentiles | 25^th^ | 1.79 | 0.93 | 4.48 | 74.2 | 24.89 | 2.88 |
|  |  |  | 75^th^ | 1.84 | 0.96 | 4.59 | 75.11 | 25.8 | 3.02 |

**p* ≤ 0.05 from the tumor-distant white matter; ** *p* ≤ 0.05 from normal (intact) cortex; *** *p* ≤ 0.05 from normal (intact) white matter. Kruskal-Wallis test. n=3-6 animals per group.
